# A White Random Laser

**DOI:** 10.1038/s41598-018-21228-w

**Published:** 2018-02-09

**Authors:** Shu-Wei Chang, Wei-Cheng Liao, Yu-Ming Liao, Hung-I Lin, Hsia-Yu Lin, Wei-Ju Lin, Shih-Yao Lin, Packiyaraj Perumal, Golam Haider, Chia-Tse Tai, Kun-Ching Shen, Cheng-Han Chang, Yuan-Fu Huang, Tai-Yuan Lin, Yang-Fang Chen

**Affiliations:** 1 Department of Physics, National Taiwan University, Taipei, 10617 Taiwan; 2 Research Center for Applied Sciences, Academia Sinica, Taipei, 10617 Taiwan; 3 Institute of Optoelectronic Sciences, National Taiwan Ocean University, Keelung, 202 Taiwan

## Abstract

Random laser with intrinsically uncomplicated fabrication processes, high spectral radiance, angle-free emission, and conformal onto freeform surfaces is in principle ideal for a variety of applications, ranging from lighting to identification systems. In this work, a white random laser (White-RL) with high-purity and high-stability is designed, fabricated, and demonstrated *via* the cost-effective materials (*e.g*., organic laser dyes) and simple methods (*e.g*., all-solution process and self-assembled structures). Notably, the wavelength, linewidth, and intensity of White-RL are nearly isotropic, nevertheless hard to be achieved in any conventional laser systems. Dynamically fine-tuning colour over a broad visible range is also feasible by on-chip integration of three free-standing monochromatic laser films with selective pumping scheme and appropriate colour balance. With these schematics, White-RL shows great potential and high application values in high-brightness illumination, full-field imaging, full-colour displays, visible-colour communications, and medical biosensing.

## Introduction

The hope of next-generation illuminants goes on the application of random laser. As an unconventional laser system, random laser is naturally endowed with two key superiorities, namely, laser-level intensity and broad-angular emissions^1–4^, which are mutually exclusive in thermal light sources, light-emitting-diodes (LEDs), and lasers.

Nowadays, white LEDs, undoubtedly, lead the mainstream lighting marketplace^5,6^. The current standard of white LEDs is dominated by the high-brightness InGaN blue LEDs integrated with wavelength-downconverting phosphors, which have been introduced the highest luminous efficacy of all white light source^7^. However, the recognized limitation in InGaN blue LEDs was not able to be overcome: A nonthermal drop in efficiency under increasing input energy density. The “efficiency drop” restricts operation only under relatively low input energy densities, which is a stark contrast with the motive for producing more photons per unit area of the LEDs chips (*i.e*., reducing the cost per lumen of the illuminations)^8^.

Lasers, with higher efficiency and output than LEDs or thermal light sources, are potential and promising alternatives^9,10^. Multi-wavelength lasers spanning the full visible spectrum have been significantly addressed in the state-of-the-art optical technologies. Previous prototypes of simultaneous red, green, and blue (RGB) emissions include a flute-type hollow-cathode He-CD laser, an Ar-Kr mixed gas laser, a cascaded optical superlattice for wavelength conversion, a combination of inorganic semiconductor laser diodes and a second harmonic Nd3þ-doped yttrium aluminum garnet (Nd: YAG), a monolithic multisegment ZnCdSSe-based semiconductor nanosheet, a single-chip dye-doped polymer, and so on^11–16^. However, there remains several significant challenges that the integration, operation, and production of nonlinear optical processes of laser modules are somewhat bulky, costly, and intricate. Besides, the constructions of conventional lasers in a single chip or a film, in which intentional gratings, waveguides, and resonators need to be precisely designed and constructed, are arduous techniques. The last and the worst, directionality, a significant nature of lasers, sets a hurdle for lighting, imaging, and display uses. These shortcomings create and promote interests in developing a strong-luminous and high-efficiency light source with competitive cost, simple design, compact size, and low spatial coherent emission.

A white random laser (White-RL) is a promising and feasible approach. Such unconventional laser lases when multiple light scattering in the disordered materials lets the gain surpass the loss^2,4^. Notably, random laser systems can generate spatially incoherent and laser-level radiation, in contrast to those existing illuminant modules (*e.g*., incandescent bulb, LEDs, superluminescent diodes, and broadband lasers). This angle-free emission is in principle ideal for not only general lighting (*e.g*., laser headlights) but also consumer products (*e.g*., spotlight, laser display, and digital laser projector) and even imaging applications (*e.g*., full-field microscopy and medical sensing). Additionally, based on the unique mechanism, random lasers hold limitless potential beyond the scope of conventional lasers, bringing new possibilities and innovative designs to advanced illuminants^3,17–20^. Since the experimental breakthrough by Lawandy *et al*.^21^, significant efforts have been made to realize random lasing actions in diversified material systems from inorganic^1^ to organic^21^ or even bio-material^22^, and within broad wavelength from ultraviolet^1^ to near-infrared^23^. These works provide strong references and theoretical foundations for the simplification of the fabrication process, minimization of cost, and miniaturization of modules, which are the primary issues for mass and large area production in the industry (*e.g*., all solution process and roll to roll printing technique)^17,24^. Notably, without the rigorous resonant cavities, the issues mentioned above are no longer an issue in the random laser systems. Moreover, wearability, flexibility, and stretchability, the three most desirable but challenging functionalities for advanced optoelectronics, are achievable in random lasers^19,25,26^. Random laser with fascinating characteristics lying somewhere between lasers and common illuminants (*e.g*., traditional light bulb and LEDs) can serve as essential foundations of laser illumination^26–34^.

In this work, a White-RL with on-chip integration based on three monochromatic organic laser dyes has been designed, fabricated, and demonstrated. The motivation to introduce organic technology into optoelectronics is because of the following irreplaceable features including low material cost, all-solution process, thin film coating, self-assembly morphology, deformability, etc^35–38^. Besides, the spectacular commercial success of organic light emitting diodes (OLEDs) or organic solar cell is another shot in the arm for scientists to exploit organic materials in other fields^39,40^. By using organic materials for lasers, we have exploited low-cost, solution-based, and self-assembled approaches to fabricate RGB monochromatic polymer films (MPFs), which can be used to generate monochromatic random lasers. These solution-processed MPFs exhibit high deformability and can be manufactured through inkjet printing technologies on arbitrary substrates^19^. Colour-tunable emission and carefully balanced White-RL are studied by the integration of three MPFs on a single chip. The results show that the emission of White-RL has high purity, stability, and angular independence, which are very important for broad illuminant applications. We highly anticipate that White-RL can be applied to lighting, imaging, display, fluorescence microscopy, biological sensing, chemical monitoring, identification, and communication^3,18–22,41^.

## Results and Discussion

**Figure 1:**
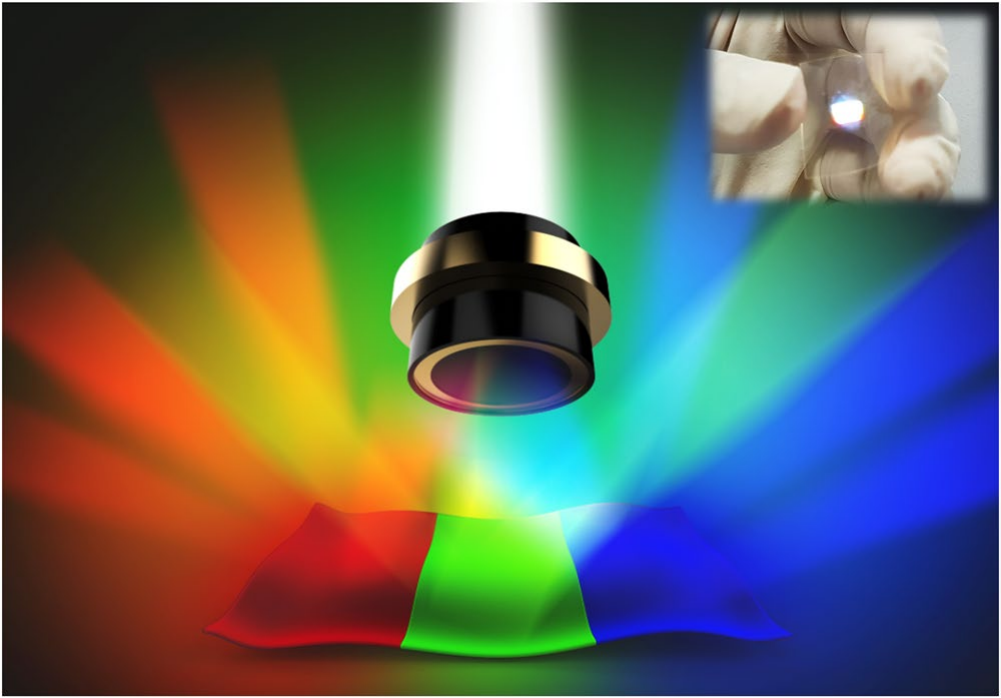
White random laser (White-RL). Schematic of the White-RL. The inset shows the real photo of the merged RGB light from the White-RL sample.

To achieve White-RL with on–chip integration for desirable optoelectronics, RGB MPFs can be synthesized separately and integrated onto arbitrary substrates as shown in Fig. 1. By individually controlling the optical excitation power on each section, the random lasing emission can run through the whole gamut of white light. These MPFs are synthesized using solution-processed and self-assembled methods, which are simple, low-cost techniques, and potentially useful for roll-to-roll printing processing and large-scale fabrication (More details of the synthesis of each MPF are provided in the Methods). The emission photo of the merged RGB light from the White-RL sample is shown in the inset of Fig. 1.

**Figure 2:**
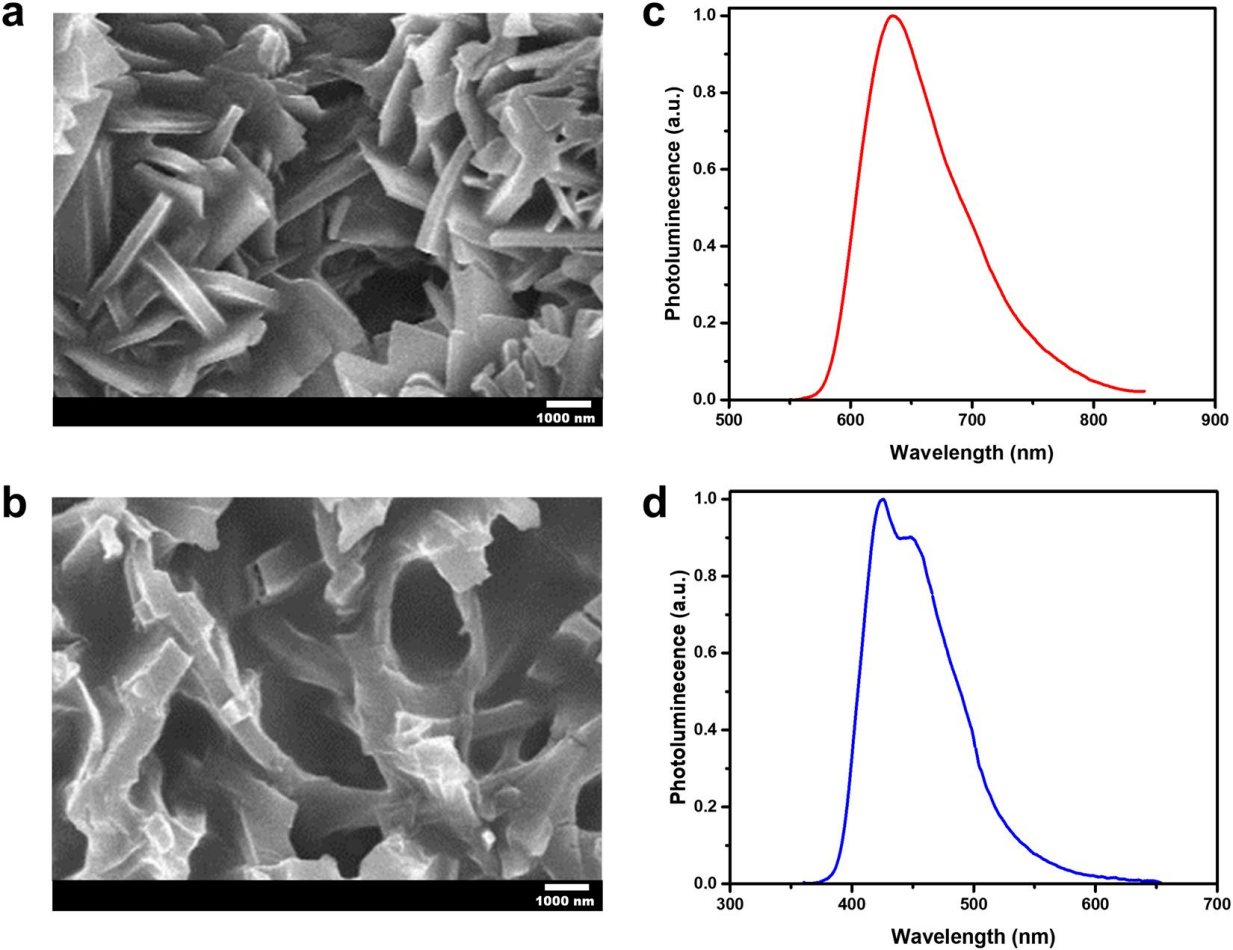
Characteristics of red (DCJTB) and blue (S420) monochromatic laser films. (**a**,**b**) Scanning electron microscopy (SEM) images of self-assembled structures of DCJTB (**a**) and S420 (**b**) doped in MPFs. (**c**,**d**) The photoluminescence spectra of DCJTB (**c**) and S420 (**d**) MPFs.

A scanning electron microscopy (SEM) image of solution-processed and self-assembled 4-(dicyanomethylene)-2-tert-butyl-6-(1, 1, 7, 7-tetramethyljulolidin-4-yl-vinyl)-4H-pyra (DCJTB) organic dye doped in red-MPF is shown in Fig. 2a. Disordered DCJTB nanoparticles and nanoplates with sizes ranging from hundreds to thousands of nanometers are observed. These nanoparticles and nanoplates are uniformly distributed over the substrate, showing various shapes. The randomly distributed DCJTB serves as a disordered gain material and scattering centers for the formation of closed feedback loops for random lasing to occur. Similarly, the SEM image of blue-MPF using stilbene-420 (S420) organic laser dye is shown in Fig. 2b. It is observed that self-aggregated disordered nanostructures are distributed and randomly doped in blue-MPF. These random nanostructures are intertwined to form complex disordered cavities. The scale length of the random cavities ranges from hundreds of nanometers to several micrometers, which is comparable with the radiation wavelength around hundreds of nanometers. It could serve as efficacious scattering centers for the formation of closed feedback loops for random lasing to occur. Besides, the refractive index contrast between the self-assembled particles of dyes covered with PMMA and air could provide sufficient condition to cause scattering. Low-magnification SEM images of red-MPF and blue-MPF are also provided in the Supplementary Information (A: Fig. S1). In addition to SEM images, the photoluminescence (PL) spectra of red and blue MPFs are shown in Fig. 2c,d. Accordingly, the broadband PL emissions are centered at around 640 nm and 460 nm, respectively.

**Figure 3:**
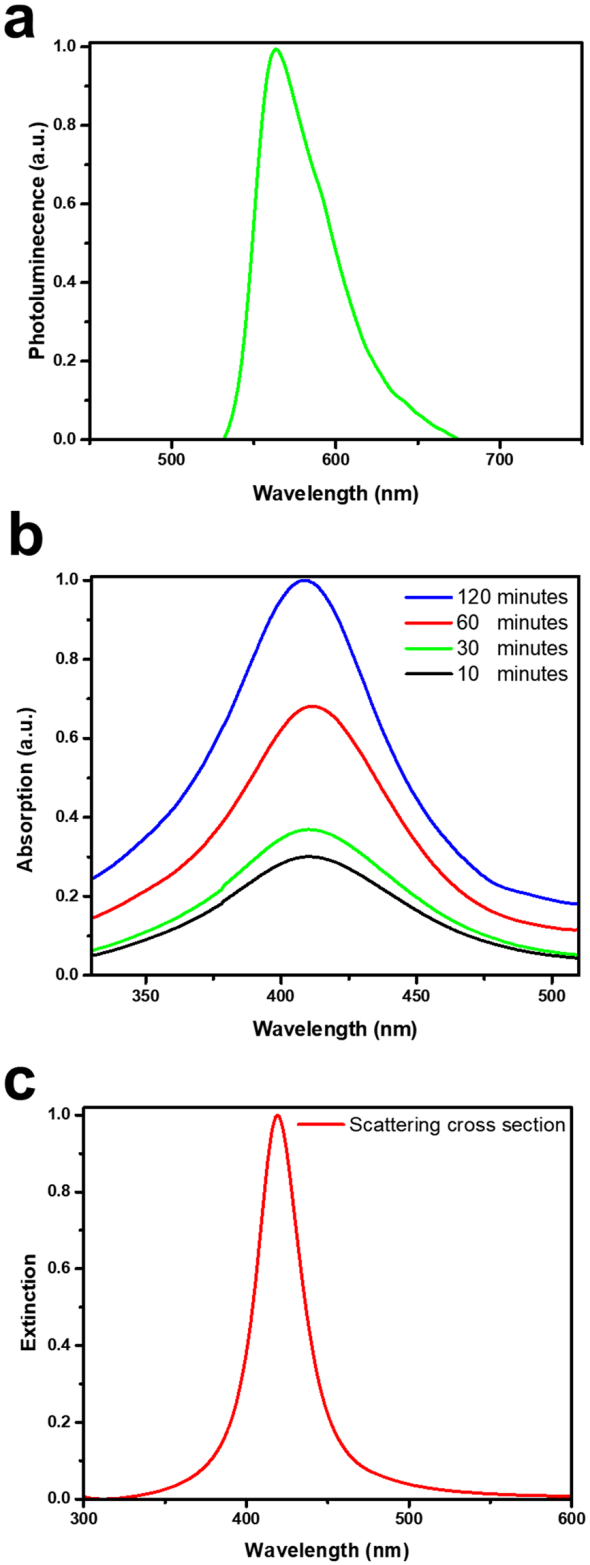
Characteristics of green (R6G) monochromatic laser film. (**a**) The photoluminescence spectra of R6G laser film. (**b**) Plasmon absorption spectra of Ag-PVA heated at 120 °C for different periods of time. (**c**) Simulation of scattering cross section of silver nanoparticles with sizes of 10 nm according to the Mie scattering theory and Drude model.

For green-MPF, although it is synthesized using rhodamine 6 G (R6G) organic laser dye. We do not find naturally formed disordered structures suitable for generating random lasing action in the R6G-doped polymer film. Instead, the green-MPF is embedded with self-assembled silver nanoparticles (Ag NPs) as plasmonic scattering centers^42^. Namely, the self-assembled Ag NPs embedded in polyvinyl alcohol (PVA) thin film *via in situ* reduction method are precipitated by thermal treatment. More details of the synthesis are provided in the Methods. Figure 3a shows the optical characteristics of R6G. The PL spectrum ranges from 540–670 nm with a peak centered at 560 nm. To confirm the existence of Ag NPs, Fig. 3b shows the difference in absorption spectra of green-MPF under different annealing time. An apparent absorption band at around 410 nm wavelength is observed and gradually increases with increasing annealing time, indicating the excitation of localized surface plasmon resonances (LSPRs) of Ag NPs in the green-MPF. A simulation curve of Ag NPs absorption using Mie scattering theory and Drude model is shown in Fig. 3c. With the radius of Ag NPs of 10 nm, the simulated absorption peak locates near 410 nm, which is in good agreement with our experimental results, indicating that the average radius of Ag NPs in green-MPF is around 10 nm. The distribution of electromagnetic field in the vicinity of Ag NPs is also simulated and provided in the Supplementary Information (B: Fig. S2) as a substantial evidence of plasmonically enhanced random lasing action dominated by Ag NPs. Moreover, the full range UV-Vis absorption spectra of the three different laser dyes are depicted in the Supplementary Information (C: Fig. S3). It shows that each absorption band covers 266 nm that led the lasing phenomena for the individual laser dyes.

**Figure 4:**
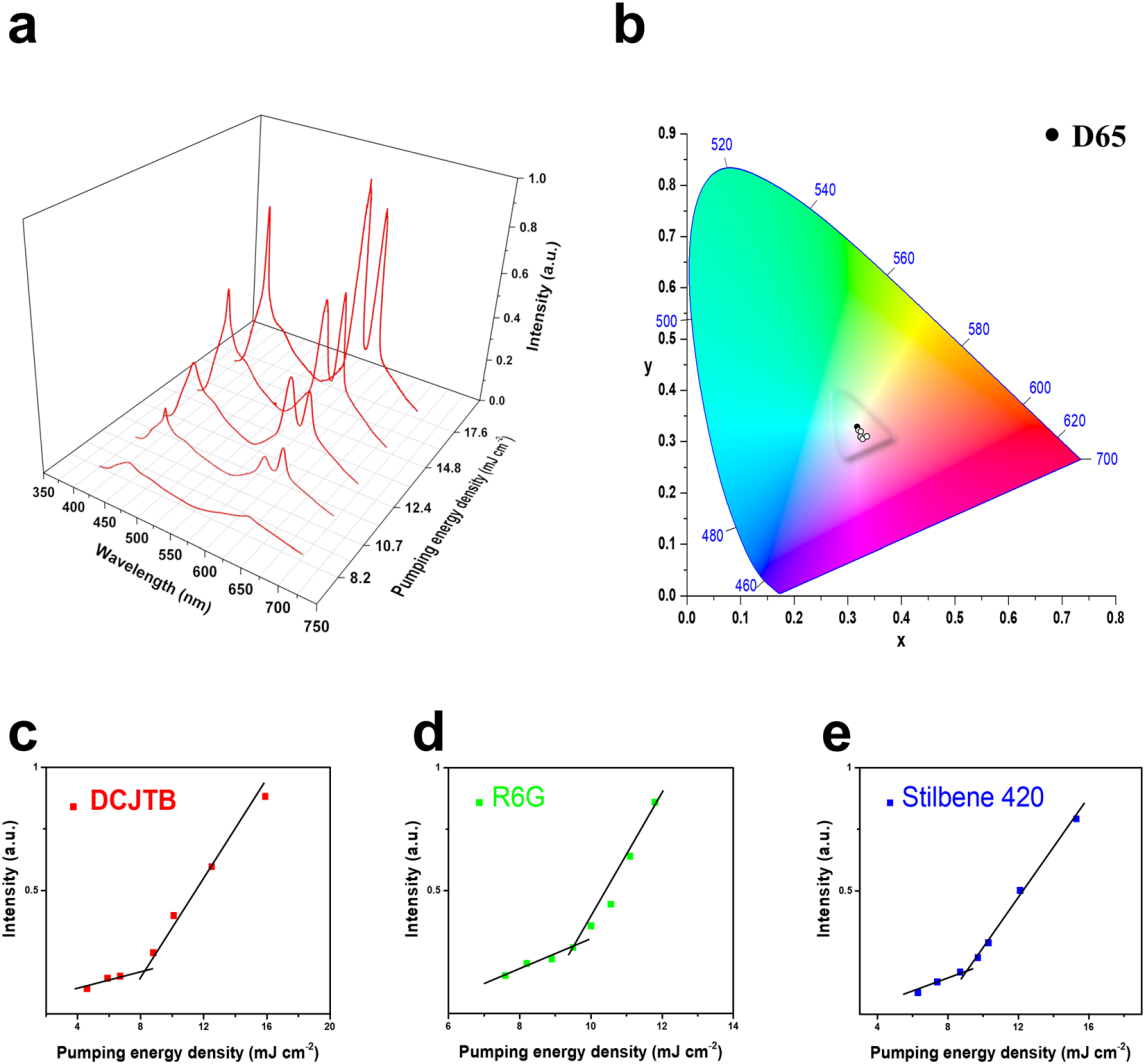
Multi-colour random laser under different pumping levels. (**a**) Multi-wavelength lasing spectra at different pumping levels (**b**) Chromaticity of the emission spectra in (**a**,**c–e**), Evolution of emission peak intensity as a function of pumping energy density of red (**c**), green (**d**), and blue (**e**) monochromatic laser films.

Typically, random lasers are characterized by a threshold in power conversion and obvious emission band narrowing above the threshold, as a result of light diffusion in disordered gain materials^43^. To study the lasing properties of MPF-based White-RL, the representative sample is optically pumped by a 266 nm pulsed laser (Supplementary Video 1). The emission spectra, chromaticity, emission intensities at different pumping energy densities are shown in Fig. 4. As the pumping energy density is below 8.2 mJ cm^−2^, only uniform and broad RGB spontaneous emissions are observed. By increasing the pumping energy density from 8.2 mJ cm^−2^ to 10.7 mJ cm^−2^, three narrowing and protruded emission bands with superlinearly increased intensity centered at approximately 620 nm, 562 nm, and 465 nm (Fig. 4a). These results suggest the possibility of the occurrence of random lasing. To have a more detailed analysis to confirm the laser action, the emission intensity of RGB MPFs as functions of 266 nm pumping energy densities are provided in Fig. 4c–e. The light-in-light-out curves show superlinear transitions from spontaneous emission regime to stimulated emission regime, which offers an excellent evidence for random lasing actions. Besides, high-resolution lasing spectra and the full width at half maximum versus the pumping energy density of RGB MPFs are performed and provided in the Supplementary Information (D: Fig. S4). The results reveal several sharp lasing peaks appear on top of the emission bands, which fluctuate randomly when pumping levels above the thresholds. The phenomenon can be identified as coherent feedback random lasers, resulting from strong interference effects introduced in disordered scattering systems^2^. We can clearly see that spontaneous emission dominates at low pumping intensities, and stimulated emission dominates at high pumping intensities. The thresholds corresponding to RGB MPFs are 8.2 mJ cm^−2^, 9.8 mJ cm^−2^, 9.1 mJ cm^−2^, respectively. To continue the exact research of lasing characteristics, the evolution of chromaticity with increasing pumping energy density is also studied and shown in Fig. 4b. Five closed circles on the CIE 1931 colour diagram represent the calculated chromaticity of White-RL under different pumping intensities, corresponding to the spectra in Fig. 4a. It is observed that with increasing pumping intensities, the chromaticity of white random lasing is almost fixed and the five circles locate closely to each other. This result suggests that the white emission is very stable and nearly unchanged in chromaticity at different pumping levels, which is very important for wide illuminant applications.

**Figure 5:**
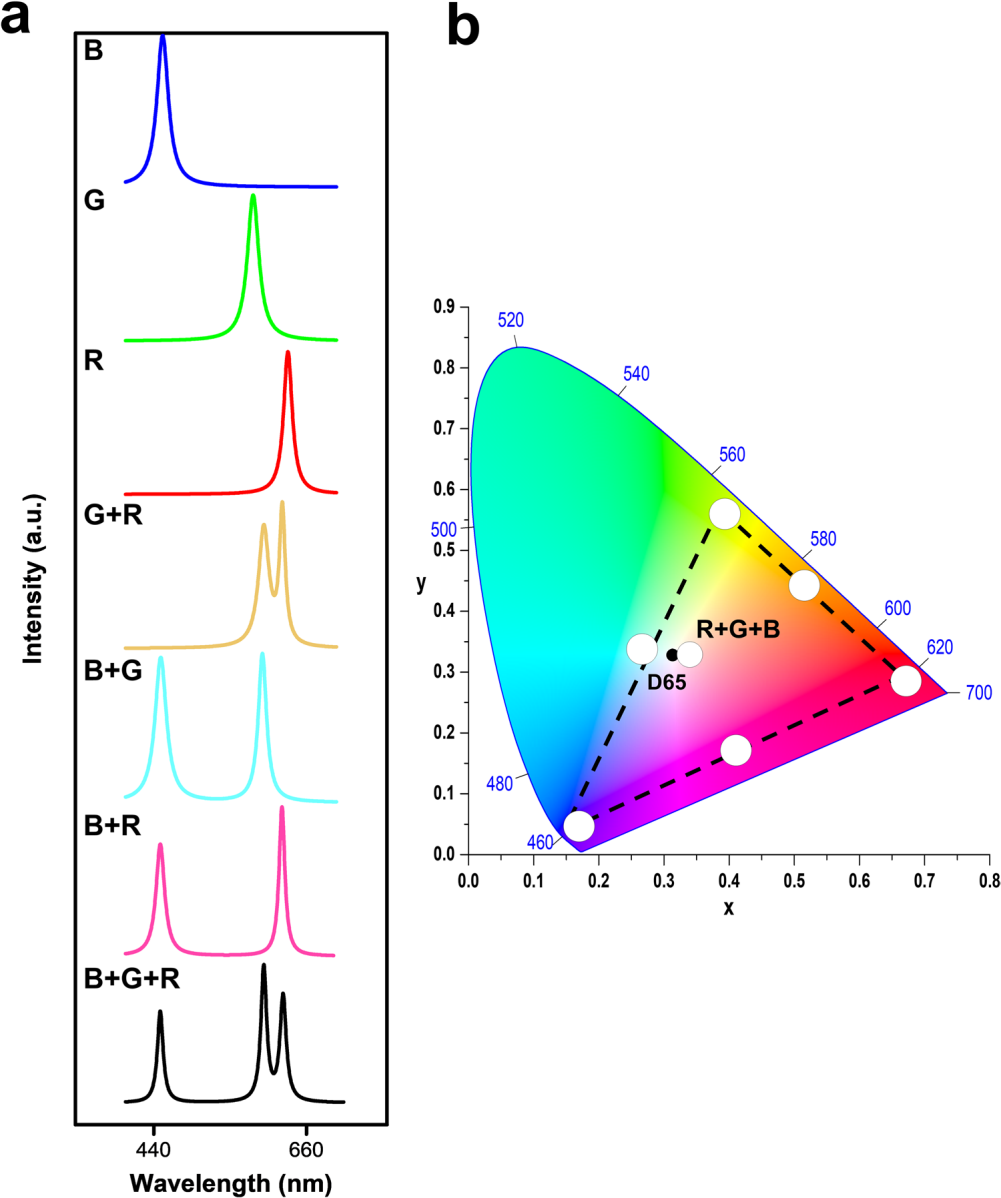
White and full-colour tunable random laser. (**a**) Lasing spectra of blue (B), green (G), red (R), red and green (R + G), green and blue (G + B), red and blue (R + B) and red, green, and blue (R + G + B) laser films. (**b**) Tunable chromaticity of the lasing spectra extracted from the spectra in (**a**).

Figure 5 demonstrates the colour-tunable lasing and white emission from the representative White-RL sample. To calculate the chromaticity of individual and mixed random lasing, the contribution of spontaneous emission is removed using Lorentz fitting method (Supplementary Information E: Fig. S5). In this way, the combined colour due to sole lasing contribution can be determined more accurately. Figure 5a and Figure S6 show the fitting spectra of individual RGB random lasing, the mixing of two-colour lasing, and simultaneous three-colour random lasing from the sample. As a result, the far-field lasing colour can be varied within a broad colour range by mixing individual RGB emission with different proportion. Figure 5b shows the calculated chromaticity of these spectra on a CIE 1931 colour diagram. It is shown that the three-elemental lasing colours form a triangle region that covers a broad range within the diagram. Therefore, colour-tunable random lasing can be achieved within this triangle gamut with appropriate mixing of the three elemental lasers, based on Grassmann’s law. Furthermore, the colour of the balanced three-colour random lasers is very close to the standard white point on the CIE 1931 diagram. This result brings us the full realization of White-RL by combining RGB MFPs on a single chip.

**Figure 6:**
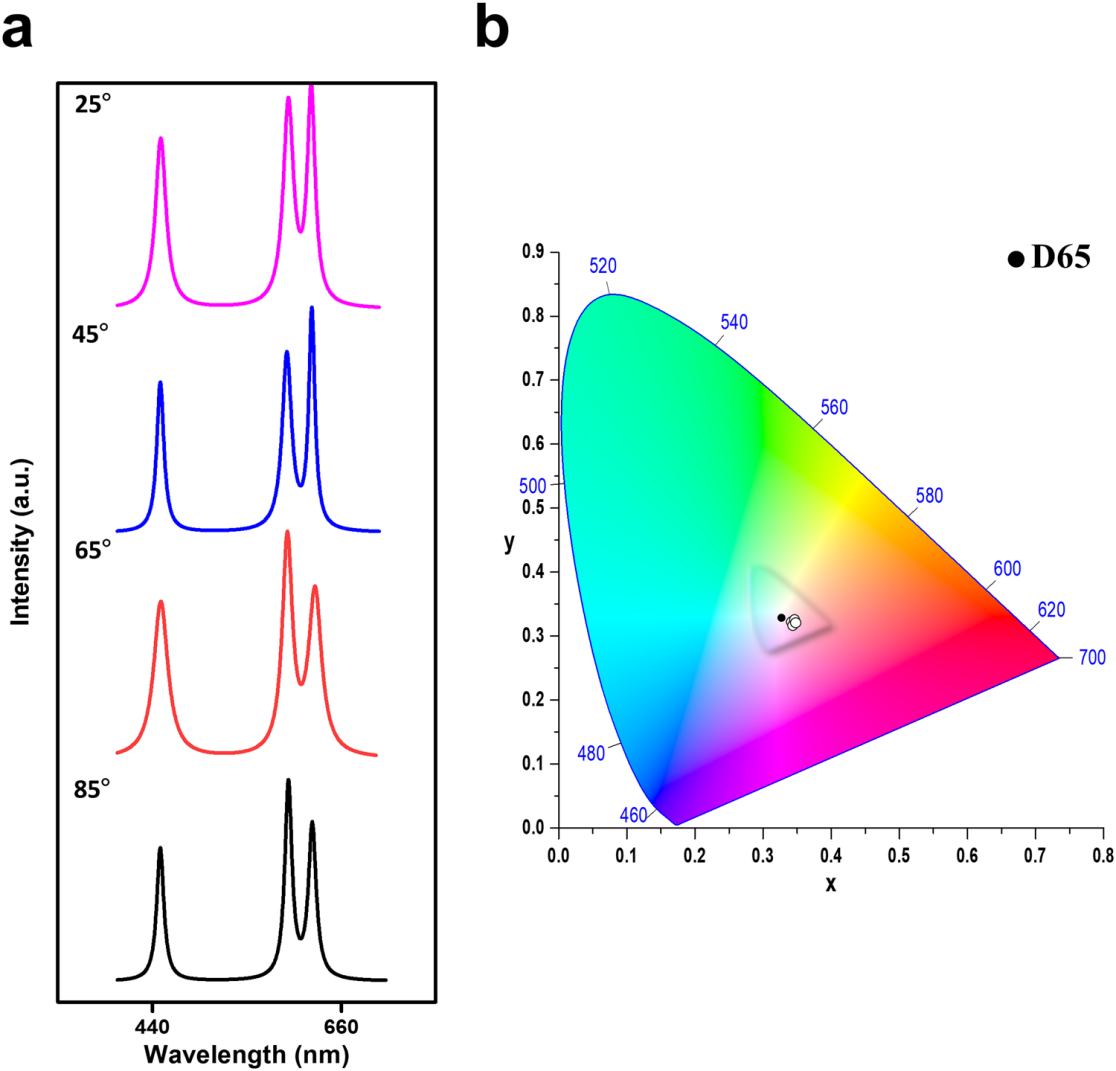
Broad angular white random laser. (**a**) Multi-wavelength lasing spectra under different observation angles. (**b**) Angle-independent chromaticity of the lasing signals extracted from the spectra in (**a**).

Figure 6 demonstrates angle-free output of MPF-based White-RL. Unlike conventional lasers, random lasers can contribute isotropic output due to random scattering in the disordered nanostructure. Figure 6a and Figure S7 show the fitting spectra of White-RL at various observation angles from θ = 25° to θ = 85° (θ refers to the angle between the normal vector of the sample and the observation direction). The wavelength, the linewidth, and the intensity of the white laser emission are almost independent of the observation angles^44^. Remarkably, in Fig. 6b, the calculated chromaticities of White-RL at different observation angles are close to the CIE standard white illuminant D65 and almost fixed in the same CIE coordinate. Furthermore, a series of photographs of white laser emission at different observation angles are also taken and provided in the Supplementary Information (G: Fig. S8). The angle-independent White-RL source can trigger disruptive innovation for cutting-edge lighting technology. In addition, the stability under ambient environment and the deformability under bending or stretching of MPF-based White-RL are also demonstrated in the Supplementary Information (H: Fig. S9–S11).

**Figure 7:**
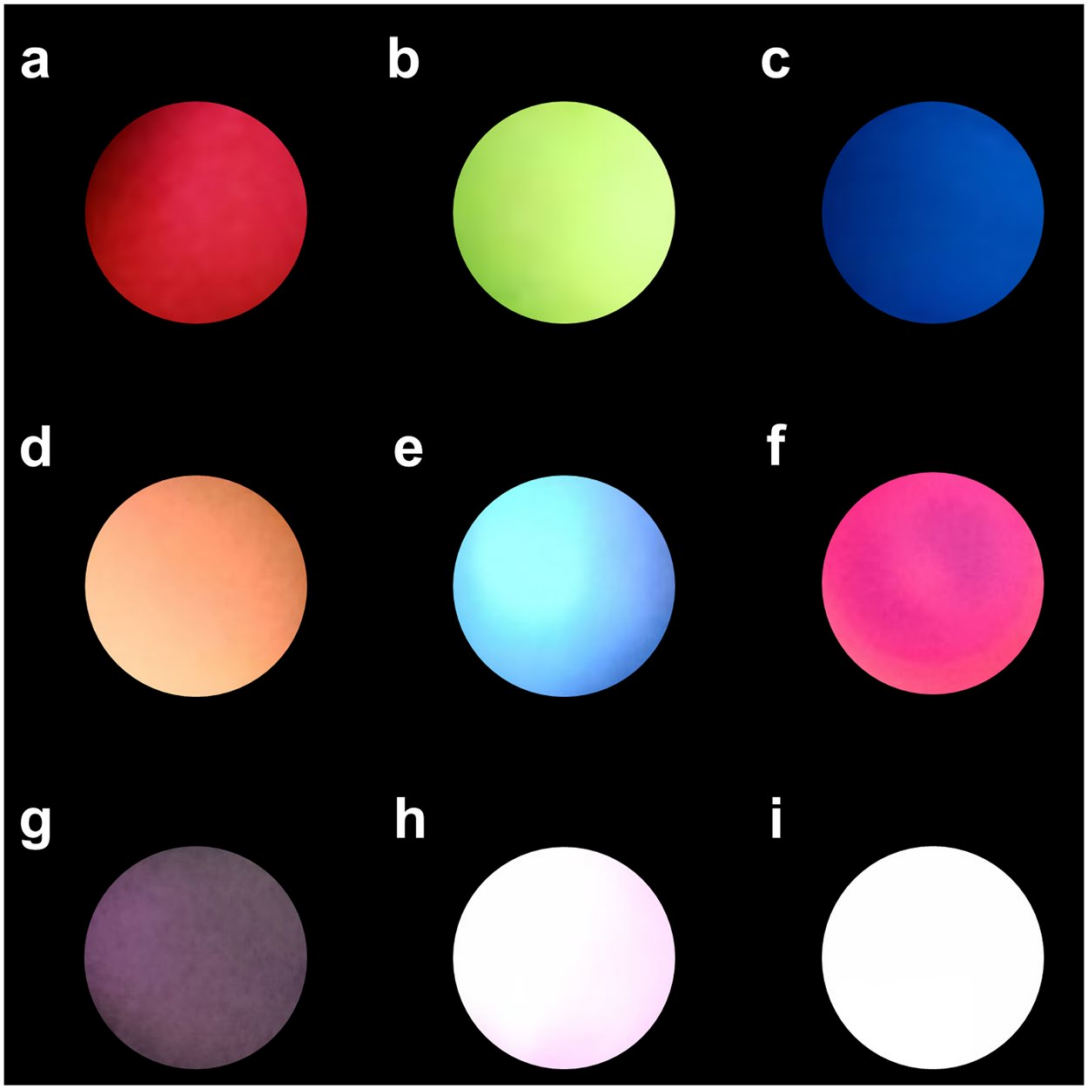
Colour tuning. (**a**–**i**) Photographs of mixed far-field emission colours red (**a**), green (**b**), blue (**c**), yellow (**d**), cyan (**e**), magenta (**f**), white emission below the threshold of pumping energy density (**g**) and above the threshold of pumping energy density (**h–i**).

To show the far-field mixing of colours with good colour rendering, we use a selective pumping method, which works as follows. First, monochromatic lasing is engendered by focusing the pumping light source to a spot on a single segment of the sample. Second, we adjust the position of the pumping spot on the chip to the junctions of two of the segments to present the two-colour mixed lasing. Finally, we focus the pumping spot on the junction of all three segments so that all three segments are simultaneously pumped to demonstrate the full white laser emission. The schematic of the selective pumping scheme is depicted in the Supplementary Information (I: Fig. S12). By selective pumping method and carefully adjusting the area of each segments being pumped, appropriate colour balance can be achieved. And, the colour of the White-RL can be broadly tuned within the gamut formed by the three monochromatic polymer laser films. Real colour images of White-RL are taken and shown in Fig. 7, corresponding to Fig. 5. Figure 7a–c show independent red, green, and blue lasing emission. Yellow, cyan, and magenta mixed lasing emissions are also achieved by selectively pumping two of the segments shown in Fig. 7d–f. Finally, in Fig. 7g–i, RGB random lasing are mixed to render as White-RL while three MPFs are simultaneously pumped with increasing pumping energy density. The photo shown in Fig. 7g is taken when the White-RL sample is optically pumped with pumping intensity below the threshold, and above the threshold (Fig. 7h,i). This colour mixing demonstration provides a practical proof-of-concept for the use of our multi-colour laser module in illumination and display applications.

Finally, to have a broader range of practical application, electrically pumped laser action is much desirable. For further perspective, the White-RL does have the electrically pumping possibility. Various organic materials, such as DCJTB, have been applied in OLED modules, serving as the light emitting layer. Moreover, there are several reports^45^, showing that a random nanostructure scattering layer can extract the light confined in OLEDs. This multiple scattering effect meets well with the concept of random lasers, and the organic laser dyes in our prototype form a strong scattering system for random lasing action to occur, significantly enhancing the possibility and feasibility to integrate with present OLED modules to achieve electrically pumping White-RL. In addition, the all-solution process exploited in this report is another plus for the development of printable OLEDs based laser devices. The White-RL shown here is the first proof-of-concept to demonstrate the laser-level and isotropic light source for illumination or imaging use.

## Conclusion

A tunable and deformable white random laser module has been successfully demonstrated. We exploit a facile and accessible method for the synthesis of self-assembled laser resonators with random sizes and chaotic distributions, providing further opportunities of random lasers for large-scale fabrication and roll-to-roll printing process. The calculated chromaticity of White-RL is not only approaching that of the CIE standard white illuminant D65 but also nearly invariable under different pumping energy densities. Remarkably, the lasing properties of White-RL (*e.g*., frequency, linewidth, intensity, and, chromaticity) are free of observation angles, which is an exclusive superiority of our white random laser system. We firmly believe that White-RL with angle-free emission, laser-level output, and deformability is an on-demand solution for the next-generation human light source, which possesses a wide range of application spanning from optical communication, lighting, display to medical sensing.

## Methods

### Sample synthesis

The glass substrates (1.5 cm × 1.5 cm) were ultrasonically cleaned for 10 min subsequently in deionized (DI) water, acetone, and isopropyl alcohol (IPA) in sequence to remove any adsorbed contaminant.

Red monochromatic polymer films (Red-MFPs) were synthesized *via* two-step solution process. The 4-(dicyanomethylene)-2-tert-butyl-6-(1,1,7,7-tetramethyljulolidin-4-yl-vinyl)-4H-pyra (DCJTB) was dissolved into dichloromethane (DCM) at room temperature with a concentration of 5 mg mL^−1^. The DCJTB solution was directly jetted on the glass substrates. Under annealing at 100 °C for 10 min, self-assembled DCJTB nanoparticles were formed. Next, the poly (methyl methacrylate) (PMMA) was spin-coated onto the DCJTB layer at a rate of 6000 rpm for 20 s to protect the nanostructures and heated at 100 °C for 10 min to dry out the PMMA. As a result, free-standing red-MPFs were formed on the glass substrates.

For the synthesis of green-MFPs, 0.6 mg Rhodamine 6 G (R6G), 0.1 mg silver nitrate (AgNO_3_), and 300 mg poly (vinyl alcohol) (PVA) were dissolved in 20 mL DI water. The solution was directly dropped on the glass substrates and heated at 120 °C for 120 min. As a result, silver nanoparticles are naturally formed in the PVA film embedded with R6G *via in situ* reduction process. Note that adding R6G in the precursor solution for the preparation of Ag nanoparticles will not dramatically affect the morphology of Ag nanoparticles as shown in the Supplementary Information (J: Fig. S13)

The synthesis of blue-MPFs is similar to that of red-MFPs, and the stilbene 420 (S420) was dissolved into ethanol at room temperature with a concentration of 10 mg mL^−1^. Then the S420 solution was directly dropped on the glass substrates and heated at 100 °C for 10 min. Next, the poly (methyl methacrylate) (PMMA) was spin-coated onto the S420 layer at a rate of 6000 rpm for 20 s to protect nanostructure and heated at 100 °C for 10 min to dry out the PMMA. As a result, free-standing blue-MPFs were formed on the glass substrates.

Finally, free-standing RGB MPFs were torn off, cut to a suitable size, and transferred to other substrates. The total thickness of each RGB MPF is around hundreds of microns. The promising fabrication area is around few square centimeters.

### Material characterization

The self-assembled structures were characterized by SEM (Hitachi S4800 microscope). The absorption spectra were measured by UV/Vis/NIR Spectrophotometer (Perkin Elmer LAMBDA 750).

### Optical measurements

To record the random lasing emission spectra, the samples were optically excited by frequency-quadrupled 266 nm pulsed Nd:YAG laser (NewWave, Tempest 300) with 4 ns pulse width and 10 Hz repetition. The energy of single pulse shot is up to 200 mJ. The pumping beam was focused into a spot of 4 mm diameter by a cylindrical lens (f = 100 mm). A bandpass filter of a 20 nm width was used to block the pump laser illumination. The emission properties were spectrally analyzed by means of a high-resolution spectrometer Jobin Yvon iHR550 with gratings of 300 and 1200 grooves/mm (spectral resolution 0.1 nm and 0.025 nm, respectively). A Synapse Thermoelectric Cooled charge-coupled device (CCD) guaranteed to −75 °C was connected to the spectroscopy software SynerJY™. All the measurements were performed at room temperature. To demonstrate the far-field mixed colour, the RGB monochromatic random laser emissions were coupled into a collimator and delivered to the camera lens directly *via* the optical fiber.

### Numerical simulation

The simulation result is performed using the commercial electromagnetic software (Lumerical). The refractive index of Ag is from the experimental data of Johnson and Christy^46^, and PVA is set as 1.5. To avoid the artificial electromagnetic waves, we set the perfectly matched layer in all the simulation boundaries.

## Electronic supplementary material

Supplementary Video

Supplementary Information
