# Supplementary material for: A White Random Laser
Source: Sci Rep. 2018 Feb 9;8:2720. doi: 10.1038/s41598-018-21228-w (PMC5807428; doi:10.1038/s41598-018-21228-w)
Supplement: Supplementary file 2 — Supplementary Information [file 41598_2018_21228_MOESM2_ESM.pdf]

Supplementary Information

A White Random Laser

Shu-Wei Chang[1‡], Wei-Cheng Liao[1‡], Yu-Ming Liao[1‡], Hung-I Lin[1], Hsia-Yu Lin[1], Wei-Ju Lin[1], Shih-Yao Lin[1], Packiyaraj Perumal[1], Golam Haider[1], Chia-Tse Tai[1], Kun-Ching Shen[2], Cheng-Han Chang[1], Yuan-Fu Huang[1], Tai-Yuan Lin[3] and Yang-Fang Chen[1]*

[1]Department of Physics, National Taiwan University, Taipei 10617, Taiwan
[2]Research Center for Applied Sciences, Academia Sinica, Taipei 10617, Taiwan
[3]Institute of Optoelectronic Sciences, National Taiwan Ocean University, Keelung 202, Taiwan
[†]These authors contributed equally to this work.
[*]e-mail: yfchen@phys.ntu.edu.tw

Table of Contents

A: Low-magnification scanning electron microscopy (SEM) images of self-assembled S420 and DCJTB 2

B: The simulation result of electric field in the vicinity of Ag nanoparticles (NPs) .. 4

C. Full range UV-Vis absorption spectra of the three different laser dyes 6

D: High-resolution lasing spectra of RGB monochromatic polymer films (MPFs) .. 7

E: Calculation of chromaticity 11

F: 3D colour-tunable laser spectra and angle-free laser spectra 13

G: Isotropic White-RL output 15

H: Stability and deformability of MPF-based white random laser (White-RL) 17

I: Schematic of the selective pumping scheme 21

J: Adding R6G after the precursor solution synthesis 22

A: Low-magnification scanning electron microscopy (SEM) images of self-assembled S420 and DCJTB

To get a better realization of the large-scale constructions, **Figure S1**a shows the low-magnification scanning electron microscopy (SEM) image of the self-assembled DCJTB nanostructures doped in red-MPF. Various particles with irregular shapes and sizes ranging from hundreds to thousands of nanometers are observed. Similarly, **Figure S1**b shows the low-magnification SEM image of the self-assembled S420 nanostructures doped in blue-MPF. Random nanostructures and cavities are observed. The length scale is around hundreds of nanometers to several micrometers. Accordingly, the emitted light is trapped within these disordered nanostructures and derives large optical gain through the recurrent scattering process, leading to the amplification of spontaneous emission and random lasing action.

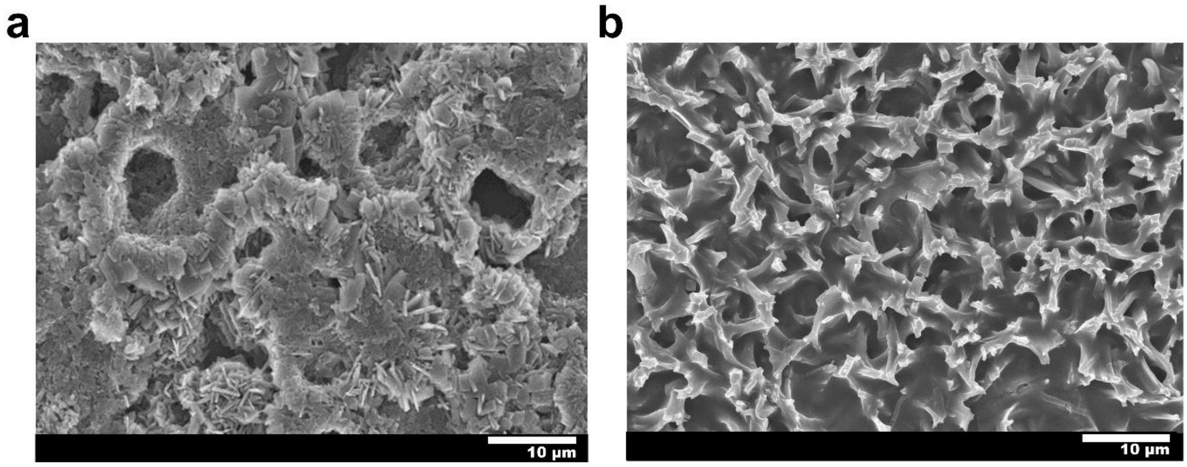

Figure S1 | Low-magnification SEM image of self-assembled DCJTB doped in red-MPF (**a**) and S420 doped in blue-MPF (**b**).

B: The simulation result of electric field in the vicinity of Ag nanoparticles (NPs)

To increase the performance, Green-MPF doped with R6G is embedded with Ag NPs as plasmonic scattering centers. Importantly, Ag NPs embedded inside PVA dominate the random lasing action in two ways. First, they provide light scattering with high efficiency. Second, the excitation of localized surface plasmon resonance enhances the local electromagnetic field near Ag NPs, enabling for high optical gain within those areas. Here we simulated the distribution of electric field intensity ($|E|^2$) at the pumping wavelength of 266 nm as shown in **Figure S2**. As expected, the distribution of $|E|^2$ around the Ag NPs can be realized as the plasmonic enhancement effect, leading to the additional scattered $|E|^2$ to excite the process of random lasing action.

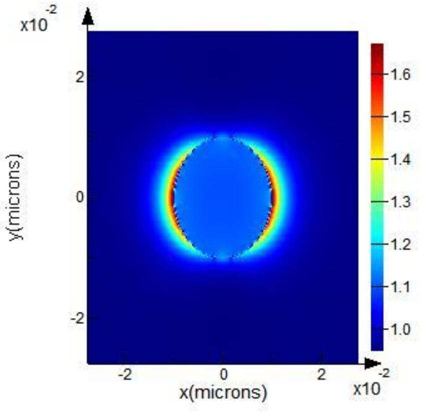

Figure S2 | Simulation of the distribution of $|E|^2$ for an Ag nanoparticle embedded inside PVA. The radius of Ag nanoparticle is 10 nm. The central emission wavelength is set at 266 nm, which corresponds to the wavelength of the incident laser.

C. Full range UV-Vis absorption spectra of the three different laser dyes

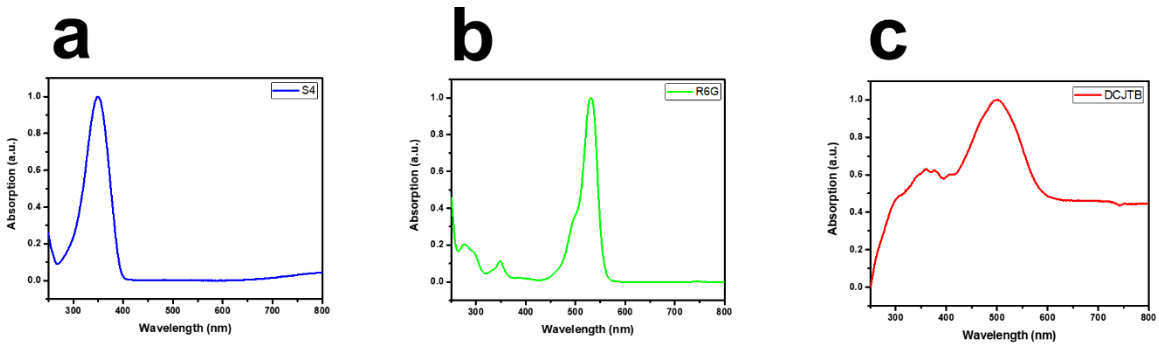

Figure S3 | Full range UV-Vis absorption spectra of stilbene 420 (**a**), R6G (**b**), and DCJTB (**c**).

D: High-resolution lasing spectra of RGB monochromatic polymer films (MPFs)

The classification of random lasers includes incoherent and coherent random lasers. Incoherent random lasers are characterized by emission band narrowing and increased emission intensity above pumping threshold. On the other hand, coherent feedback random lasers are characterized by random sharp lasing peaks that emerge on top of the emission bands. The mechanisms for coherent and incoherent random lasers are different. Incoherent feedback random lasing can be described by light diffusion in disordered gain medium, while for coherent feedback random lasers, strong interference effects should be considered. Sharp lasing peaks appear due to randomly-formed cavities that provide multiple scattering of light, enabling for strong interference along the cavity paths.

Our proposed system can be identified as a coherent feedback random laser. However, it is quite hard to be observed in the low-resolution lasing spectra (grating 300 grooves/mm with spectral resolution 0.1 nm) since the lasing signals from different modes may be averaged out in low-resolution detection. Thus, we provided the high-resolution lasing spectra (grating 1200 grooves/mm with spectral resolution 0.025 nm) as shown in **Figures S4**a-c. It is found that all the spectra display similar lasing behavior. Below the threshold excitation intensity, the photoluminescence spectrum is a single broad spontaneous emission peak. While the pumping power increased just above the threshold, multiple narrow peaks emerged in the emission spectrum. At higher pumping power well above threshold, the discrete peaks became even

sharper and stronger than those observed under lower pumping power. The full width of half maximum (FWHM) versus the pumping energy density is also presented. With a more detailed examination, the position of the wavelength, number, and the intensity of discrete laser peaks with extremely narrow linewidth changed randomly. The above-mentioned results are typical and well-known characteristics of coherent feedback random lasing action. Moreover, **Figures S4**d-f show the obvious narrowing of the full width at half maximum (FWHM) when the exciting energy surpasses the threshold, which is a fundamental property of laser action.

Coherent feedback random laser may provide more efficient and stronger laser emission since the strong scattering system can trap the coherent photons to form a closed loop in the disordered nanostructures. In this type of random lasing system, the interference effect occurs in a closed loop. Its typical characteristic is the laser spikes fluctuating within the spectral emission. Researchers now attempt to control the coherent random lasing modes. Mode-locking and single-mode random lasers have been demonstrated by the pumping scheme, Raman gain, intentional defect sites, and bioinspired photonic structure. On the other hand, incoherent feedback random laser can take place under dexterous scattering system, where the photons can partially return to the gain media but not to the original position. Thus, there is no spatial resonance. Since the mean frequency of the random laser only depends on the frequency of the emission band of the gain media. Such relatively broad spectral characteristics (as much as few to tens nanometer wide) can find various applications. For example, with low temporal

coherence, it can truly reduce speckle noise, serving as a promising speckle-free laser-level light source for imaging or sub-micron optical lithography.

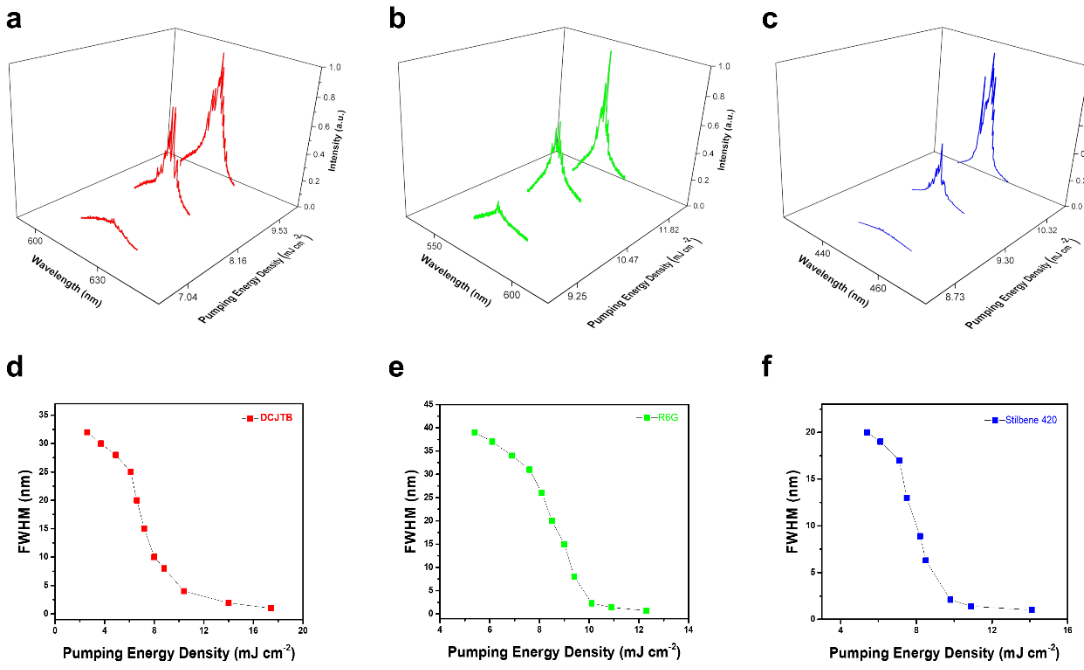

Figure S4 | High-resolution emission spectra of red (**a**), green (**b**), and blue (**c**) MPFs. Random sharp peaks are observed in high pumping intensities, indicating typical coherent random lasing. The full width at half maximum (FWHM) as a function of pumping energy density of red (**d**), green (**e**), and blue (**f**) MPFs.

E: Calculation of chromaticity

To calculate the chromaticity of white-RL, the random lasing signals and spontaneous emission are decomposed using Lorentz fitting method as shown in **Figure S5**. Subsequently, the unnecessary influence from spontaneous emission is eliminated, and the pure RL emission, which is of our interests, is studied in Figures 5, 6, S6, S7, S10, and S11. As a result, the chromaticity of White-RL is calculated using these fitted spectra to emphasize the variation of pure RL. It is shown that the chromaticity of White-RL is very close to ideal white emission and nearly independent of observation angles.

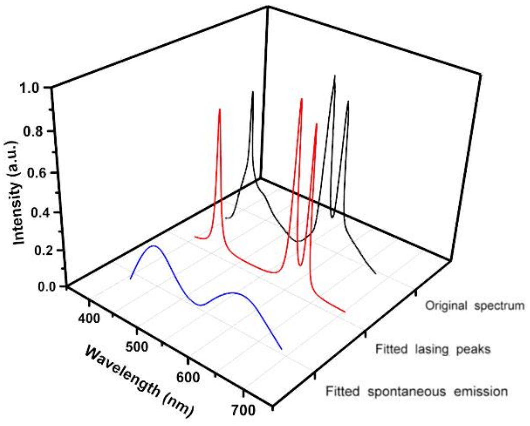

Figure S5 | Original emission spectra, the fitted spontaneous emission, and fitted lasing spectra using Lorentz fitting method.

F: 3D colour-tunable laser spectra and angle-free laser spectra

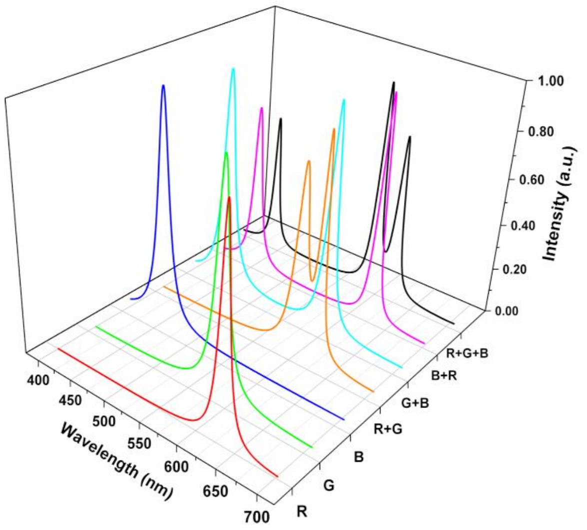

Figure S6 | 3D lasing spectra of blue (B), green (G), red (R), red and green (R+G), green and blue (G+B), red and blue (R+B) and red, green, and blue (R+G+B) laser films.

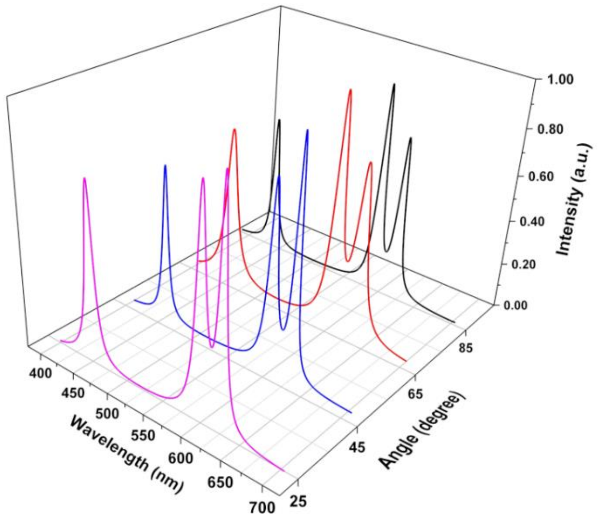

Figure S7 | 3D multi-wavelength lasing spectra under different observation angles 25°, 45°, 65°, and 85°.

G: Isotropic White-RL output

Omnidirectional emission is a specific property of random lasers, as a result of multiple scattering of light in disordered systems. Real images of far-field White-RL emission in different observation angles are collected and shown in **Figure S8**. Random lasing with ideal white emission can be observed at different directions covering 4π solid angles with stable emission intensity.

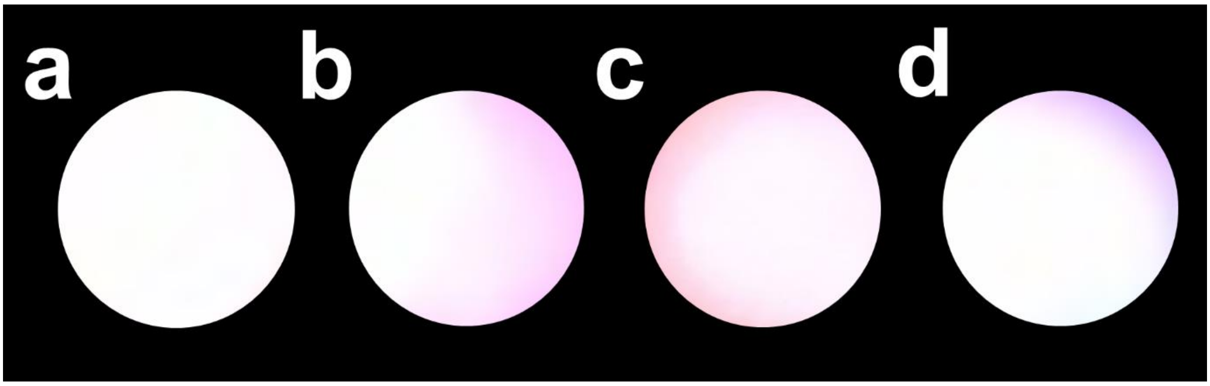

Figure S8 | Real photos of White-RL at different observation angles of 25° (**a**), 45° (**b**), 65° (**c**), and 85° (**d**).

H: Stability and deformability of MPF-based white random laser (White-RL)

The samples are exposed to the air for different days to test the stability of organic laser dye shown in **Figure S9**. Differences in intensities are hardly observed, showing that MPF-based White-RL has great stability under ambient condition, which is very important for practical applications. On the other hand, the lasing performance of the dyes may be weakened under exposure to high energy UV pulses pumping. In our sample synthesis, we do concern this issue. The poly (methyl methacrylate) (PMMA) or poly (vinyl alcohol) (PVA) thin film was spin-coated onto RGB monochromatic films to protect laser dyes from possible damage and thus prolong the lifetime of White-RL.

Moreover, deformability remains a key factor towards the development of portable and wearable optoelectronic devices. To demonstrate the deformability of MPF-based White-RL, lasing spectra of White-RL with and without external strain are performed and shown in **Figure S10** where the applied strain is defined by the change in device dimension with respect to its original size. There are little differences in the spectra when the sample is stretched under 30% of external strain. The bendability of the device is estimated by recording the device performance at a bending diameter of 10 cm as shown in **Figure S11**. The emission spectrum of the sample under bending is similar to that of non-bending one. Therefore, MPF-based White-RL exhibits high deformability, which is potentially useful for advanced optoelectronics.

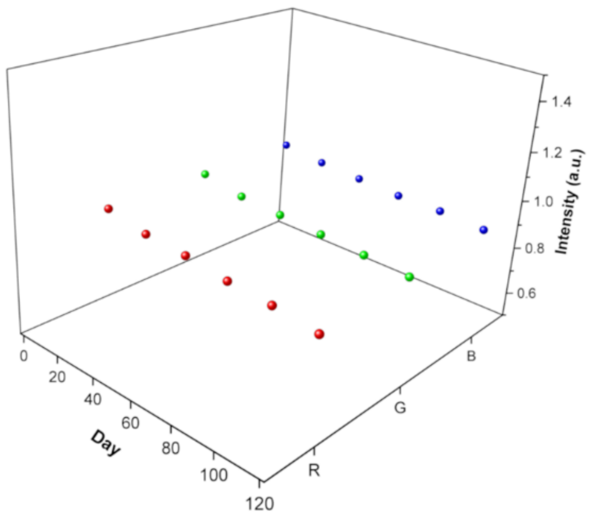

Figure S9 | Lasing intensities of red, green, and blue colours as a function of different days.

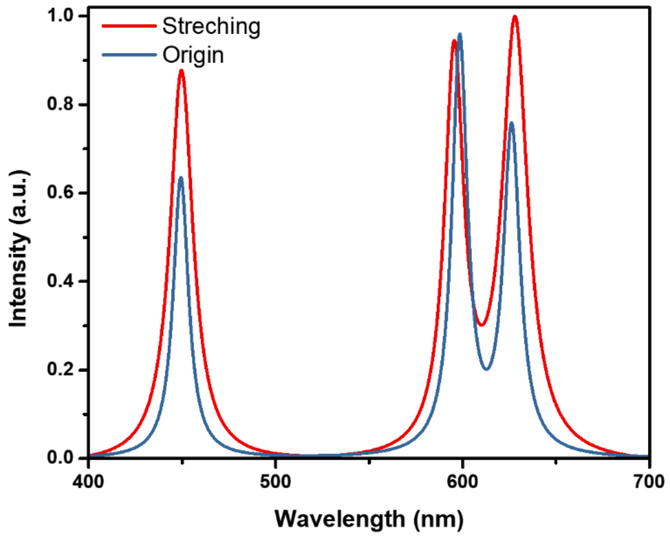

Figure S10 | White-RL lasing spectra measured with and without being stretched.

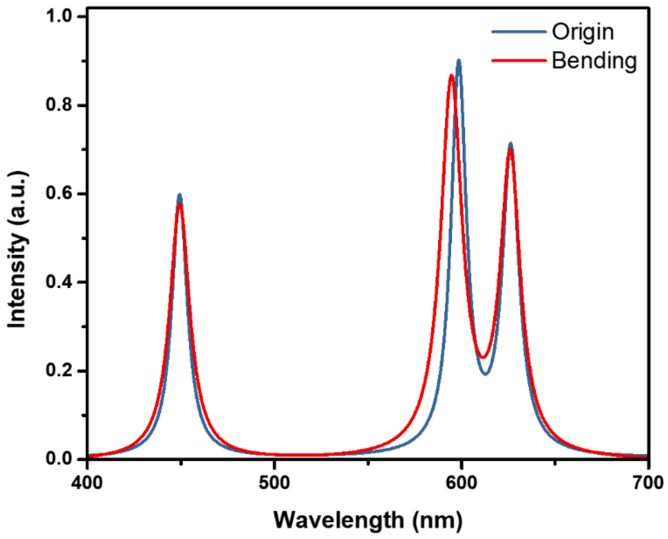

Figure S11 | White-RL lasing spectra measured with and without being bent.

I: Schematic of the selective pumping scheme

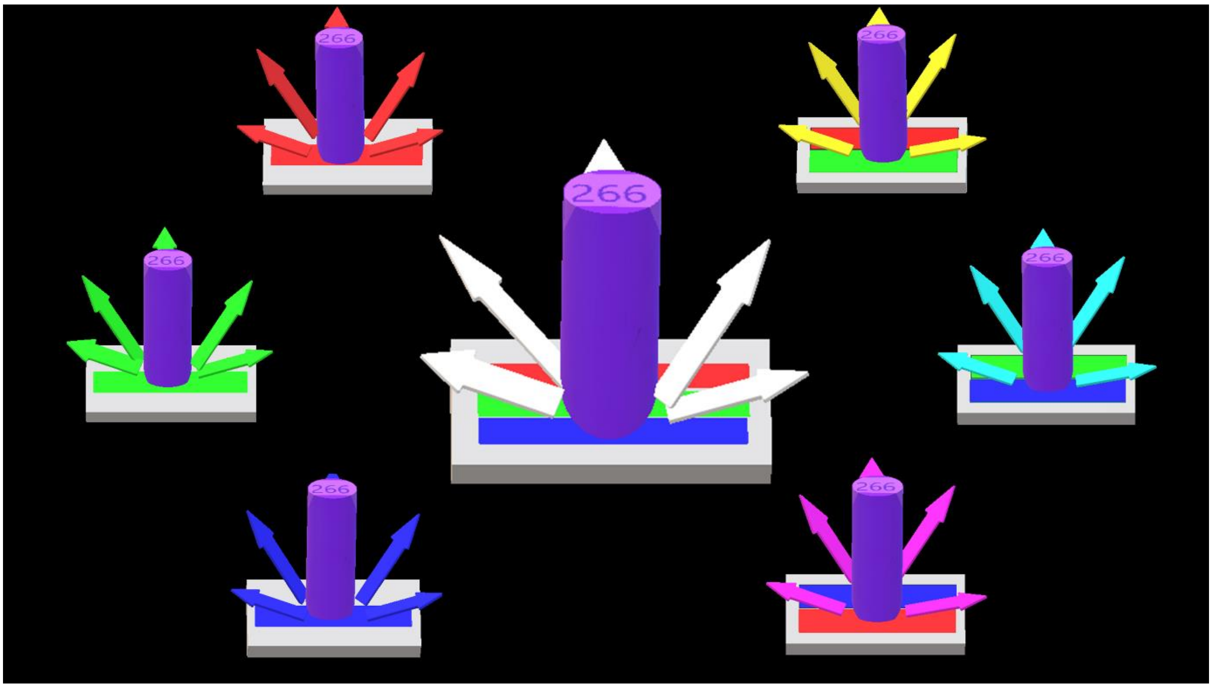

Figure S12 | The schematic of the selective pumping scheme.

J: Adding R6G after the precursor solution synthesis.

Figure S13a shows the absorption spectra of two comparing fabrication process: (1) Adding R6G after precursor solution; (2) Adding R6G before precursor solution synthesis. Both absorption spectra present a similar absorption band at around 410 nm wavelength, indicating the excitation of localized surface plasmon resonances (LSPRs) of Ag NPs. Furthermore, we use this new fabrication process (adding R6G after precursor solution) on the green monochromatic polymer film synthesis to examine the lasing performance. In **Figure S13b**, the light-in-light-out curves indicate that there is no obvious threshold change compared with Figure 4d. Based on these results, we think that there is no dramatic morphology change of Ag nanoparticles under two different processing schemes.

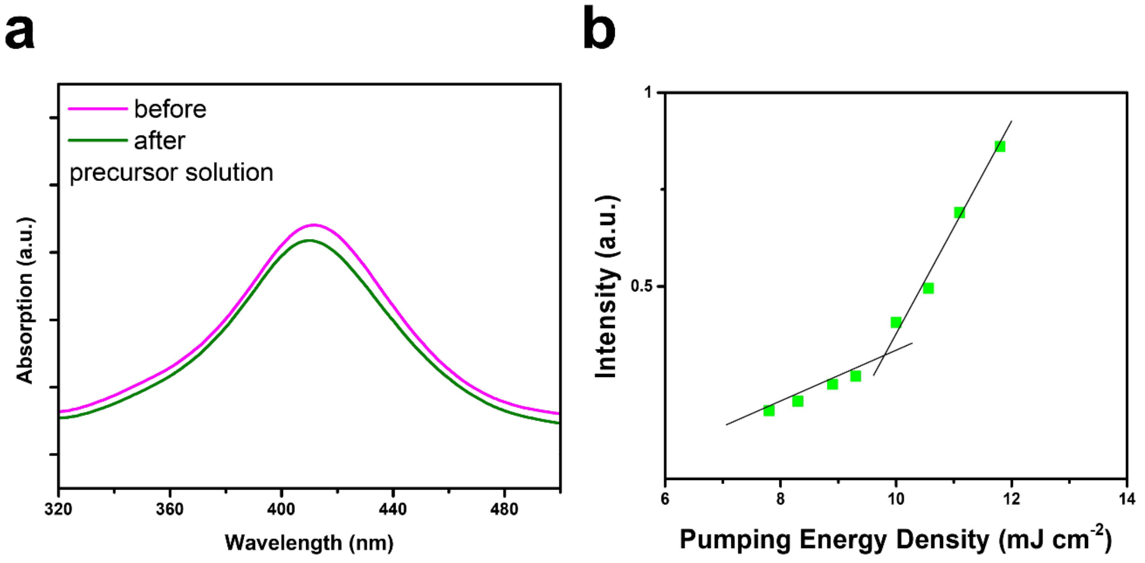

Figure S13 | a, Plasmon absorption spectra of Ag-PVA. Adding R6G after precursor solution synthesis (Green line). Adding R6G before precursor solution synthesis (Red line). **b,** Evolution of emission peak intensity as a function of pumping energy density of green monochromatic laser film. (adding R6G after precursor solution).
